# Supplementary material for: Gastrodin and Gastrodigenin Improve Energy Metabolism Disorders and Mitochondrial Dysfunction to Antagonize Vascular Dementia
Source: Molecules. 2023 Mar 13;28(6):2598. doi: 10.3390/molecules28062598 (PMC10059574; doi:10.3390/molecules28062598)
Supplement: Supplementary file 1 [file molecules-28-02598-s001.zip › molecules-2183375-supplementary.pdf]

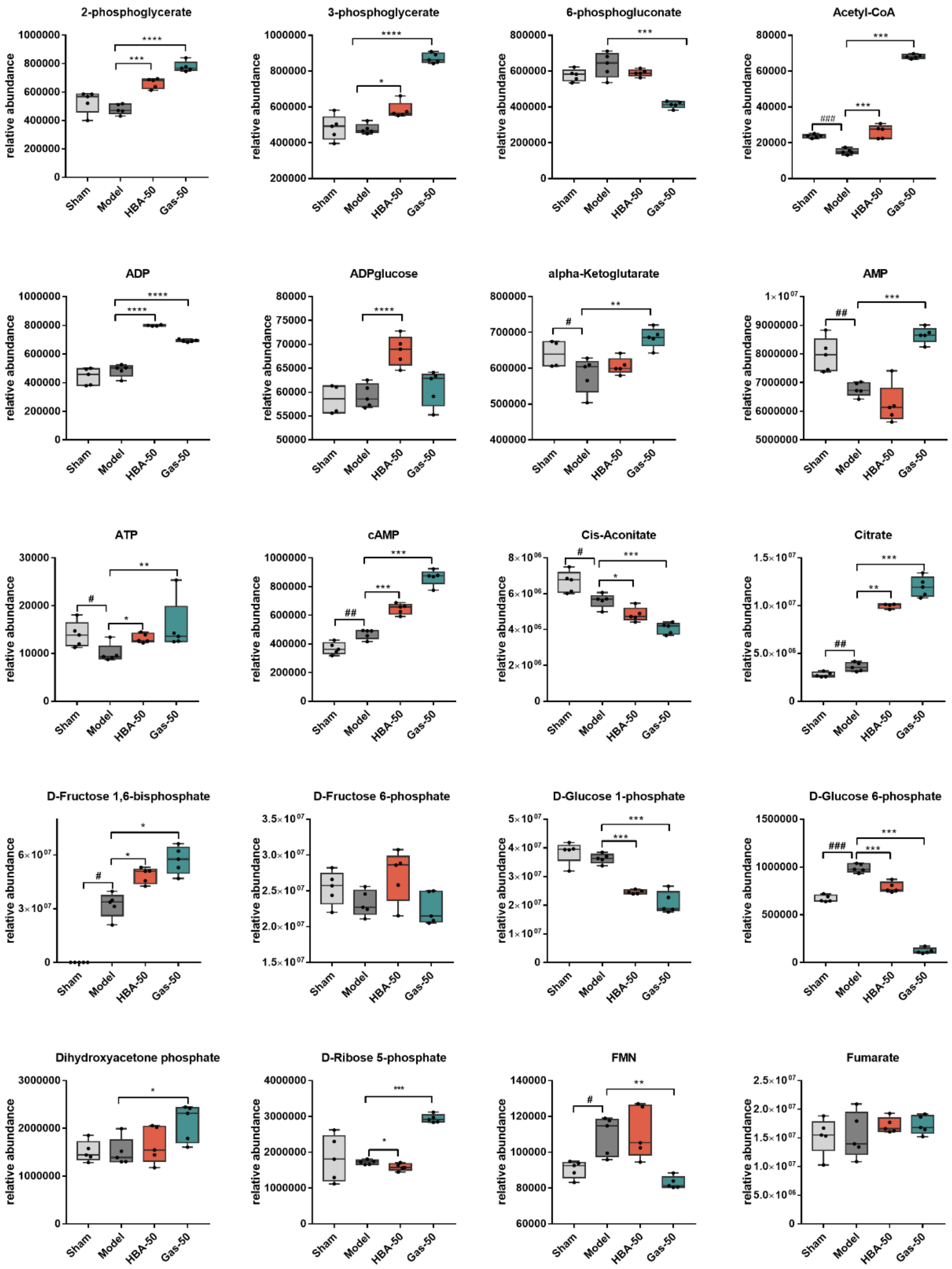
2-phosphoglycerate
3-phosphoglycerate
6-phosphogluconate
Acetyl-CoA
ADP
ADPglucose
alpha-Ketoglutarate
AMP
ATP
cAMP
Cis-Aconitate
Citrate
D-Fructose 1,6-bisphosphate
D-Fructose 6-phosphate
D-Glucose 1-phosphate
D-Glucose 6-phosphate
Dihydroxyacetone phosphate
D-Ribose 5-phosphate
FMN
Fumarate
relative abundance
Sham
Model
HBA-50
Gas-50

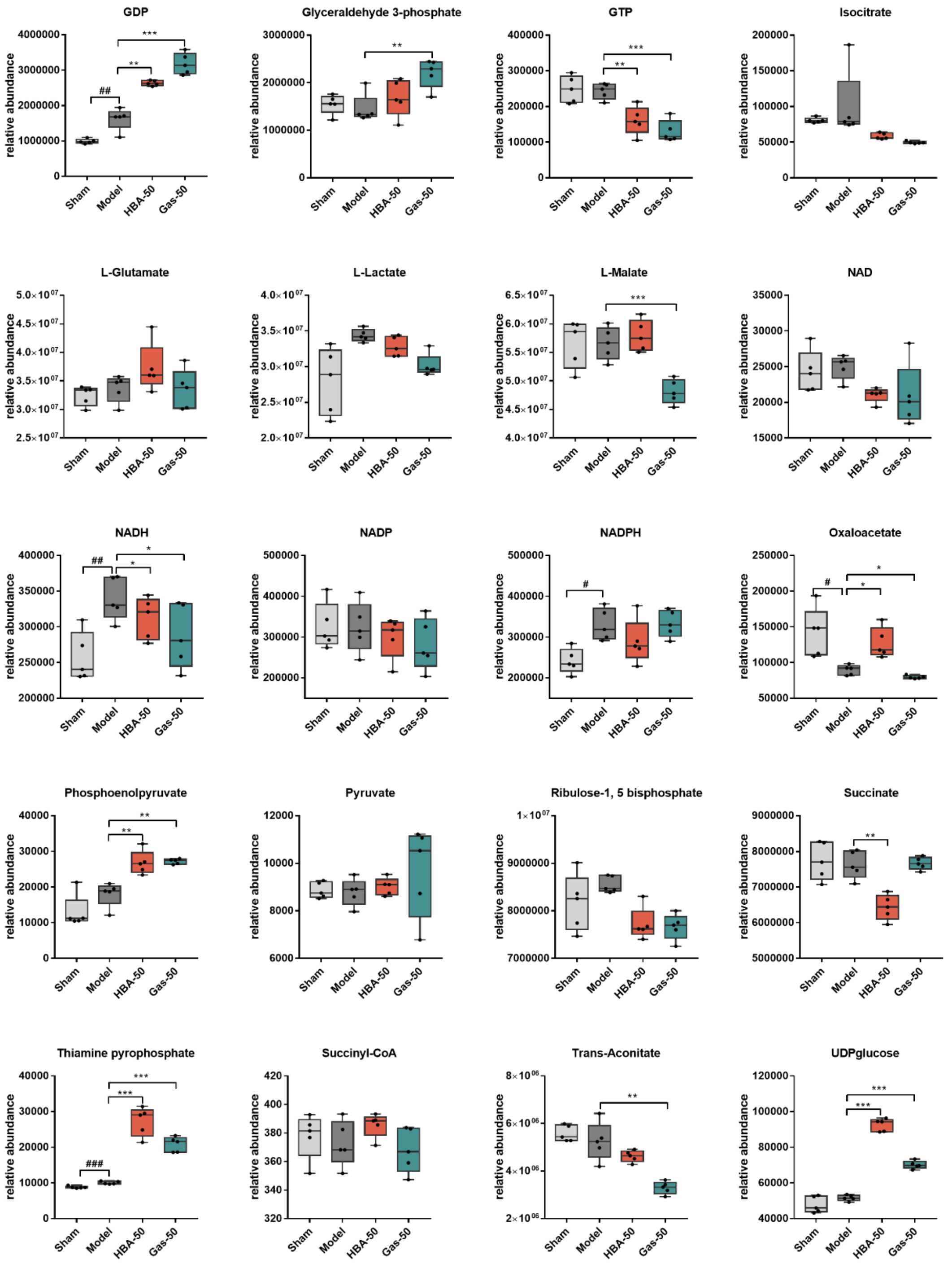

Figure S1. Expression trends of the 40 metabolites in each group. (ANOVA, $n = 5$). Sham = white, Model = gray, HBA-50 = red, Gas-50 = green. Boxplot: boxplot medians (center lines), interquartile ranges (box ranges), whisker ranges. $^{\#}P< 0.05$, $^{\#\#}P< 0.05$, $^{\#\#\#}P< 0.001$ vs Sham, $^{*}P< 0.05$, $^{**}P< 0.01$, $^{***}P< 0.001$ vs Model.
